# Divergent SARS-CoV-2 variant emerges in white-tailed deer with deer-to-human transmission

**DOI:** 10.1038/s41564-022-01268-9

**Published:** 2022-11-10

**Authors:** Bradley Pickering, Oliver Lung, Finlay Maguire, Peter Kruczkiewicz, Jonathon D. Kotwa, Tore Buchanan, Marianne Gagnier, Jennifer L. Guthrie, Claire M. Jardine, Alex Marchand-Austin, Ariane Massé, Heather McClinchey, Kuganya Nirmalarajah, Patryk Aftanas, Juliette Blais-Savoie, Hsien-Yao Chee, Emily Chien, Winfield Yim, Andra Banete, Bryan D. Griffin, Lily Yip, Melissa Goolia, Matthew Suderman, Mathieu Pinette, Greg Smith, Daniel Sullivan, Josip Rudar, Oksana Vernygora, Elizabeth Adey, Michelle Nebroski, Guillaume Goyette, Andrés Finzi, Geneviève Laroche, Ardeshir Ariana, Brett Vahkal, Marceline Côté, Allison J. McGeer, Larissa Nituch, Samira Mubareka, Jeff Bowman

**Affiliations:** 1 National Centre for Foreign Animal Disease, Canadian Food Inspection Agency, Winnipeg, Manitoba Canada; 2 Department of Veterinary Microbiology and Preventative Medicine, College of Veterinary Medicine, Iowa State University, Ames, IA USA; 3 Department of Medical Microbiology and Infectious Diseases, University of Manitoba, Winnipeg, Manitoba Canada; 4 Department of Biological Sciences, University of Manitoba, Winnipeg, Manitoba Canada; 5 Faculty of Computer Science, Dalhousie University, Halifax, Nova Scotia Canada; 6 Department of Community Health & Epidemiology, Dalhousie University, Halifax, Nova Scotia Canada; 7 Shared Hospital Laboratory, Toronto, Ontario Canada; 8 Sunnybrook Research Institute, Toronto, Ontario Canada; 9 Wildlife Research and Monitoring Section, Ontario Ministry of Natural Resources and Forestry, Peterborough, Ontario Canada; 10 Ministère des Forêts, de la Faune et des Parcs, Quebec City, Quebec Canada; 11 Public Health Ontario, Toronto, Ontario Canada; 12 Department of Microbiology & Immunology, Western University, London, Toronto, Ontario Canada; 13 Canadian Wildlife Health Cooperative, Ontario-Nunavut, Department of Pathobiology, University of Guelph, Guelph, Ontario Canada; 14 Public Health, Health Protection and Surveillance Policy and Programs Branch, Ontario Ministry of Health, Toronto, Ontario Canada; 15 Centre de Recherche du CHUM, Montréal, Quebec Canada; 16 Département de Microbiologie, Infectiologie et Immunologie, Université de Montréal, Montréal, Quebec Canada; 17 Department of Biochemistry, Microbiology and Immunology, University of Ottawa, Ottawa, Ontario Canada; 18 Ottawa Institute of Systems Biology, University of Ottawa, Ottawa, Ontario Canada; 19 Centre for Infection, Immunity, and Inflammation, University of Ottawa, Ottawa, Ontario Canada; 20 Sinai Health System, Toronto, Ontario Canada; 21 Department of Laboratory Medicine and Pathobiology, University of Toronto, Toronto, Ontario Canada; 22 Environmental and Life Sciences Graduate Program, Trent University, Peterborough, Ontario Canada

**Keywords:** SARS-CoV-2, Evolution

## Abstract

Wildlife reservoirs of broad-host-range viruses have the potential to enable evolution of viral variants that can emerge to infect humans. In North America, there is phylogenomic evidence of continual transmission of severe acute respiratory syndrome coronavirus 2 (SARS-CoV-2) from humans to white-tailed deer (*Odocoileus virginianus*) through unknown means, but no evidence of transmission from deer to humans. We carried out an observational surveillance study in Ontario, Canada during November and December 2021 (*n* = 300 deer) and identified a highly divergent lineage of SARS-CoV-2 in white-tailed deer (B.1.641). This lineage is one of the most divergent SARS-CoV-2 lineages identified so far, with 76 mutations (including 37 previously associated with non-human mammalian hosts). From a set of five complete and two partial deer-derived viral genomes we applied phylogenomic, recombination, selection and mutation spectrum analyses, which provided evidence for evolution and transmission in deer and a shared ancestry with mink-derived virus. Our analysis also revealed an epidemiologically linked human infection. Taken together, our findings provide evidence for sustained evolution of SARS-CoV-2 in white-tailed deer and of deer-to-human transmission.

## Abstract (web summary)

White-tailed deer are a potential reservoir of SARS-CoV-2 variants.

## Main

Human-pathogenic coronaviruses, such as severe acute respiratory syndrome coronavirus (SARS-CoV), SARS-CoV-2 and Middle East respiratory syndrome coronavirus, are likely to have been transmitted to humans either directly from reservoir wild animal hosts (for example, horseshoe bats) or through intermediate hosts such as civets or camels^1–6^. Emergence of human-pathogenic coronaviruses may result in sustained human-to-human transmission, with ongoing viral evolution, as has been observed with SARS-CoV-2 and the emergence of variants, including variants of concern (VOCs). Viral diversity may also occur from inter-species transmission and evolution in new hosts, as was observed during human–mink–human transmission of SARS-CoV-2 (ref. ^7^).

The evolution of divergent viral lineages can affect immunology, biology and epidemiology of any virus, as well as altering detection, vaccine efficacy, disease severity and transmission. Virus evolution can impact individual and population health. For example, the SARS-CoV-2 Omicron variant had 59 genome-wide mutations when it emerged, including 37 in the spike protein, which resulted in reduced efficacy of vaccines to the original emergent strain due to antibody evasion^8^. There are several competing hypotheses for the evolution of Omicron, with one hypothesis considering it may have evolved after SARS-CoV-2 transmission from humans to rodents (or another, as yet unidentified, animal reservoir^9^) and back into humans again (‘spillback’)^10^.

As of September 2022, observational and experimental reports in free-living, captive, domestic and farmed animals have shown that SARS-CoV-2 can infect at least 54 non-human mammalian species^11–14^. The high degree of similarity of the primary SARS-CoV-2 host cell receptor, human angiotensin converting enzyme 2, among mammals may explain the broad host-range of SARS-CoV-2 (ref. ^15^). Zooanthroponosis (reverse zoonotic disease transmission) has been documented in outbreaks of SARS-CoV-2 among farmed mink (*Neovison vison*)^16,17^ and pet hamsters (*Mesocricetus auratus*)^18^. The Netherlands experienced outbreaks of SARS-CoV-2 in mink farms^19^, and whole genome sequencing (WGS) provided evidence for the emergence of a ‘cluster 5’ variant among farmed mink with a unique combination of mutations, and identified spillback from mink to humans^19^. These mutations raised concerns about the potential to erode vaccine efficacy, contributing to a decision in Denmark to cull mink^20^. Similarly, the finding of SARS-CoV-2 in pet hamsters led Hong Kong authorities to cull thousands of animals^21^.

There have been suggestions (based on experimental data for VOCs) that SARS-CoV-2 host cell receptor tropism has expanded over time, increasing concerns about the potential for spillover into animals. For example, the Alpha variant can infect mice (*Mus musculus*), and the Omicron variant spike glycoprotein can bind to avian ACE2 receptors, unlike ancestral SARS-CoV-2 (refs. ^22,23^). This underscores the potential for ongoing expansion of susceptible host species as VOCs continue to emerge.

The white-tailed deer (*Odocoileus virginianus*) is a common North American ungulate that is susceptible to SARS-CoV-2. An experimental study reported that deer develop subclinical infection^24^. A subsequent study found that 40% of free-ranging deer sampled in Michigan, Illinois, New York and Pennsylvania, United States were positive for SARS-CoV-2 antibodies^25^. Transmission of SARS-CoV-2 among deer, and multiple spillovers from humans to deer, have also been reported^26–28^. So far, most of the SARS-CoV-2 isolated and reported in deer has been similar to lineages circulating among humans in the same region, which has led to proposal of multiple, recent, spillover events^27,28^. Also, a divergent Alpha-like virus has been reported in deer, providing some evidence of sustained transmission in deer^29^. Persistence of SARS-CoV-2 in wild animals might conceivably result in viral evolution through adaptation to the animal host. Although virus isolates that are circulating in animals may become less fit for humans, a risk of future emergence into the human population, with unknown consequences, remains.

In this Article, to assess the extent of infection in white-tailed deer, the potential for a deer reservoir of SARS-CoV-2 and the risk of deer-to-human transmission, we initiated a SARS-CoV-2 surveillance programme of white-tailed deer in Ontario, Canada.

## Results

### Divergent SARS-CoV-2 found in deer

**Fig. 1:**
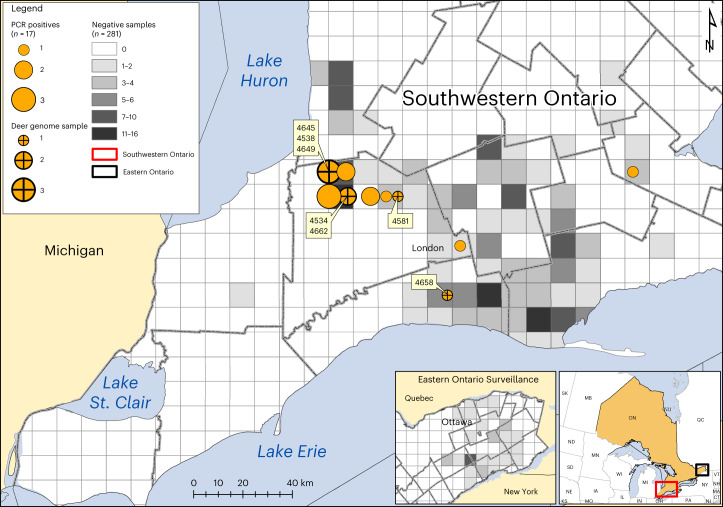
SARS-CoV-2 in white-tailed deer sampled in Ontario, 2021. Circle size indicates the relative number of positive white-tailed deer (*n* = 17/298), with crosses showing samples from which viral genomes were recovered (*n* = 7). Four-digit genomic sequence ID labels are shown in yellow boxes. Location of negative samples are indicated using grey as per the legend. The detailed map depicts Southwestern Ontario (red rectangle on inset map). SARS-CoV-2 RNA was not detected in samples from Eastern Ontario.

From 1 November to 31 December 2021, 300 white-tailed deer were sampled from Southwestern (*N* = 249, 83%) and Eastern (*N* = 51, 17%) Ontario, Canada during the annual hunting season (Fig. 1). The majority of sampled white-tailed deer were adults (94%), with comparable numbers of females (*N* = 135, 45%) and males (*N* = 165, 55%). We collected 213 nasal swabs and tissue from 294 retropharyngeal lymph nodes (RPLN), which were tested for SARS-CoV-2 RNA using reverse transcription polymerase chain reaction (RT–PCR).

Five of 213 (2.3%) nasal swabs were positive by two independent RT–PCR analyses at separate institutes (untranslated region (UTR) and envelope (E) RT–PCR targets Ct <40; and E and N2 RT–PCR targets Ct <36). Sixteen RPLN were also confirmed by PCR. Overall, SARS-CoV-2 RNA was detected in 21 samples representing 6% (17/298) of hunter-harvested white-tailed deer; all positive animals were adult deer from Southwestern Ontario and the majority (65%) were female (Fig. 1 and Supplementary Table 1). Two deer were excluded from further analysis owing to indeterminate RPLN results with no corresponding nasal swab.

To determine viral lineage and potentially infer key epidemiological relationships, we sequenced three high-quality SARS-CoV-2 consensus genomes from the five positive nasal swabs using a standard amplicon-based approach. All samples were also independently extracted and sequenced using a capture-probe-based approach for confirmation. By combining the amplicon and capture-probe sequencing data, five high-quality genomes (white-tailed deer nasal swabs: 4581, 4645, 4649, 4658 and 4662) and two partial genomes (white-tailed deer RPLNs: 4538 and 4534) were recovered (Supplementary Table 1). The samples were negative for human RNAse P by PCR and the majority (median 79.7%; Supplementary Table 1) of non-SARS-CoV-2 reads mapped to the white-tailed deer reference genome, demonstrating that contamination from human-derived SARS-CoV-2 sequences was highly unlikely.

**Fig. 2:**
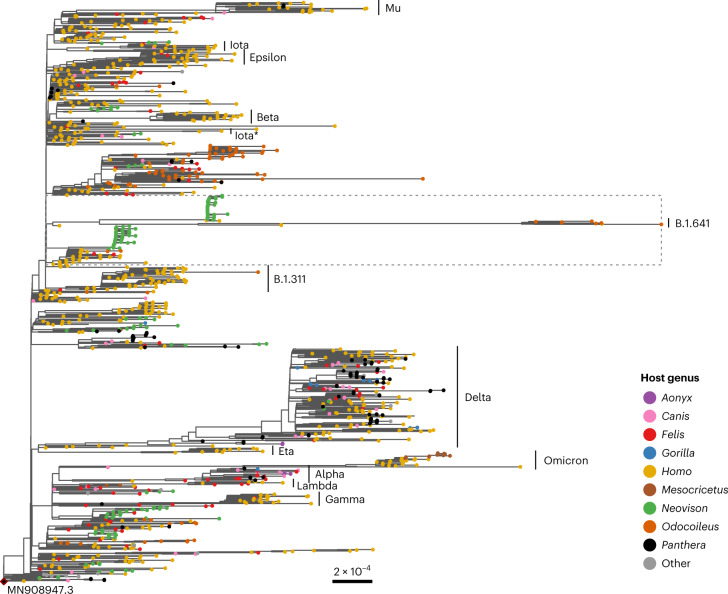
ML phylogeny of white-tailed deer viral genomes. Included are Ontario deer-derived genomes and an associated human sample (which have been collectively designated as lineage B.1.641) and a representative sample of the global diversity of human and animal-derived SARS-CoV-2 (*n* = 3,645). This phylogeny represents all non-human animal-derived samples (with the exception of domestic mink from Europe, which were subsampled) in GISAID at the time of sampling along with a representative subsample of human-derived genomes. VOCs and variants previously designated as variants under investigation within the tree are annotated and nodes are coloured by host genus (as indicated in the legend). The dotted line indicates the samples selected for the local ML analysis (Fig. 3).

**Extended Data Fig. 1:**
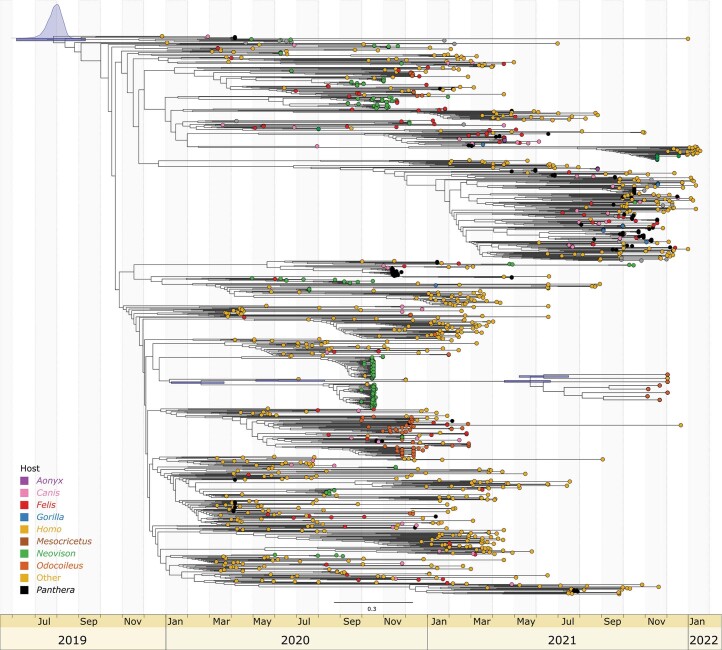
A BEAST inferred time tree of B.1.641. The tree was estimated using a HKY + G4 substitution model, log-normal distributed clock rates based on a strict molecular clock of 9.5 × 10^−4^ (inferred from tip-to-root regression) and a constant-sized coalescent. Analysis was performed used a fixed topology derived from the global ML analysis with internal node heights (and root height) inferred by BEAST. The 95% HPD node bars are shown in blue for the tree root and selected nodes of interest.

Maximum-likelihood (ML) and parsimony-based phylogenetic analyses were used to examine lineage origins and relatedness, and showed that these deer-derived viral genomes formed a highly divergent clade within the B.1 PANGO lineage/20C Nextstrain clade (100% ultrafast bootstrap (UFB)) that shared a most recent common ancestor (MRCA) between May and July 2021 (95% highest posterior density (HPD) node age bounds; Extended Data Fig. 1). The B.1 lineage encompasses substantial diversity and was the genetic backbone from which the Beta VOC, Epsilon and Iota variants and notable mink (*Neovison*) outbreaks emerged (Fig. 2). The Ontario deer lineage forms a very long branch with 76 conserved nucleotide mutations relative to ancestral SARS-CoV-2 (Wuhan Hu-1) and 49 relative to their closest common ancestor with other genomes in GISAID (as of March 2022). The closest branching genomes in GISAID are human-derived sequences from Michigan, United States, sampled approximately 1 year prior (November/December 2020), which were inferred to share an MRCA with the Ontario deer lineage between May and August 2020 (95% HPD node age bounds; Extended Data Fig. 1). These human-derived sequences in turn are closely related to a mixed clade of human and mink sequences from Michigan collected in September and October 2020. The Ontario white-tailed deer lineage has been designated as PANGO lineage B.1.641.

Given the distorting effects of long-branch attraction and incomplete sampling, there is a degree of uncertainty in the phylogenetic placement of the white-tailed deer samples. However, the geographical proximity (Michigan is adjacent to Southwestern Ontario) and the mix of human and other animal cases (for example, human and mink cases) provide compelling evidence supporting this placement. Given the degree of divergence and potential for phylogenetic biases, we conducted three analyses to examine the possibility of recombination. Using 3Seq^30^, bolotie^31^ and HyPhy’s^32^ genetic algorithm recombination detection method^33^ with datasets representative of human and animal SARS-CoV-2 diversity in GISAID (as of March 2022) there was no indication of recombination within, or having given rise, to this lineage.

### Potential deer-to-human transmission

**Fig. 3:**
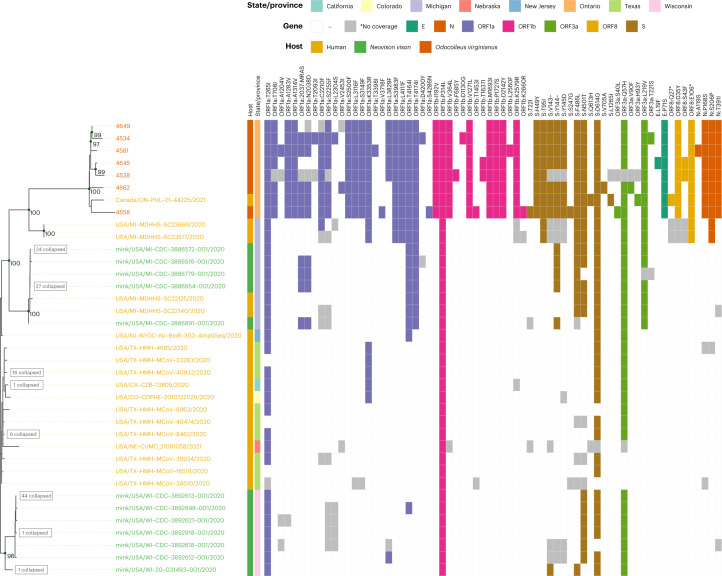
Phylogeny of the B.1.641 Ontario white-tailed deer lineage. Genomes are annotated with the presence/absence of amino acid mutations relative to SARS-CoV-2 Wuhan Hu-1. Genomes were selected to characterize the relationship between Ontario white-tailed deer samples, the related Ontario human sample and closest B.1 human and mink samples from Michigan, United States (dotted segment in Fig. 2). Internal nodes in the phylogeny are annotated with UFB values ≥95%, and leaves with identical amino acid profiles were collapsed as indicated. Host species for each sample is shown by the leaf label colour and first annotation column as per the legend, with geographic location in the second annotation column. Amino acid mutations are coloured by corresponding gene, with grey indicating sites that were too poorly covered to determine presence/absence (for example, sites in partial deer-derived genomes 4538 and 4534).

Our phylogenetic analysis also identified a human-derived sequence from Ontario (ON-PHL-21-44225) that was highly similar (80/90 shared mutations; Supplementary Table 2) and formed a well-supported monophyletic group (100% UFB) with the white-tailed deer samples (Fig. 3). The small number of samples and relative diversity within B.1.641 make it difficult to determine the exact relationship between the human sample and the white-tailed deer samples (78% UFB for a MRCA with 4,658). However, global (Fig. 2) and local (Fig. 3) ML analyses and an UShER-based^34^ (Supplementary Fig. 1) parsimony analysis all support this human sample belonging to B.1.641.

The human-derived viral sequence also has a plausible epidemiological link to the white-tailed deer samples since it was collected in the same geographical region (Southwestern Ontario), during the same time period (autumn 2021). The human case had known close contact with deer in the week before symptom onset and had no known contact with any individuals that had tested positive for SARS-CoV-2 before or after contact with deer. At the time of the human case detection, the Ontario COVID-19 Genomic Network aimed to sequence 100% of eligible confirmed PCR-positive SARS-CoV-2 samples collected from human cases, and no other genetically related human-derived samples were identified. It should be noted that not all requested human samples are successfully sequenced, and the Omicron surge necessitated a reduction in the human-derived SARS-CoV-2 sampled for sequencing in Ontario in late 2021 (ref. ^35^).

### Zoonosis-associated mutations

**Fig. 4:**
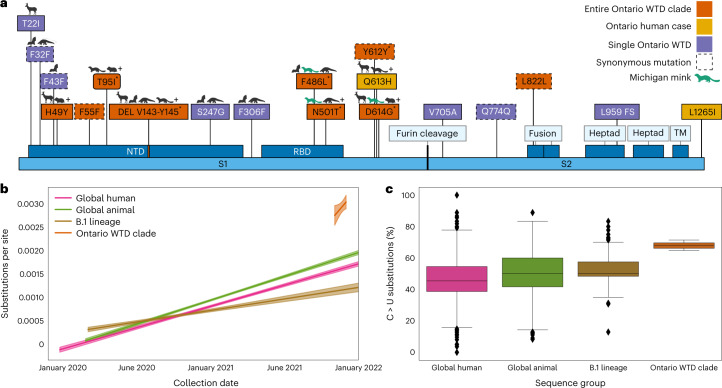
Evolution of the B.1.641 Ontario white-tailed deer lineage. Evolution of the B.1.641 lineage is presented relative to other animal-derived genomes, the ancestral B.1 lineage and the global SARS-CoV-2 diversity. **a**, Spike mutations present within the Ontario white-tailed deer (WTD) lineage. Amino acid changes present in all five Ontario WTD sequences and associated human case (orange); only in the human sample (yellow); and only in a single WTD genome (purple). Animal symbols indicate mutations in bat-, deer-, pangolin- and hamster-derived SARS-CoV-2 sequences. ‘+’ indicates presence of the mutation in additional non-human animal species, and green indicates those in Michigan mink samples. ‘*’ indicates spike mutations that were inferred to have originated subsequent to the divergence of B.1.641 from their MRCA with the Michigan-derived human samples. Spike annotations were derived from UniProt P0DTC2 (DEL, deletion; FS, frameshift; TM, transmembrane; RBD, receptor binding domain; NTD, N-terminal domain) and are not shown to scale. A complete list of mutations from across the entire genome can be found in Supplementary Table 2. **b**, Root-to-tip regression analysis based on the representative SARS-CoV-2 diversity in the global ML phylogeny (Fig. 2). Substitutions per site per year trends (and 95% confidence intervals) from ordinary least squares regression analyses are shown for all human samples (0.9 × 10^−3^ to 1.0 × 10^−3^), animal-derived samples (1.0 × 10^−3^ to 1.1 × 10^−3^), the B.1 lineage (0.4 × 10^−3^ to 0.6 × 10^−3^) and the Ontario WTD clade (0 to 8 × 10^−3^). **c**, Consensus substitutions (%) corresponding to a change from a reference C allele to an alternative U allele. Boxes represents the 25% quartile, median and 75% quartile, with error bars capturing the minimum and maximum values within 1.5× interquartile range. This was calculated from consensus sequences across a subsample of global human SARS-CoV-2 diversity (earliest and most recent genomes from each PANGO lineage, *n* = 3,127), global animal diversity (all animal genomes in GISAID at time of sampling, *n* = 1,522), B.1 lineage (all genomes assigned to this lineage in GISAID as of January 2022, *n* = 206) and B.1.641.

Using the five high-quality, complete deer-derived sequences and related human-derived sequence, we analysed the prevalence of mutations across GISAID in general as well as within VOC and animal-derived samples (Supplementary Table 2) to identify and contextualize key mutations. Of the 76 mutations shared among the 6 high-quality B.1.641 sequences, 51 are in ORF1ab (with 11 and 9 each in Nsp3 and Nsp4, respectively) and 9 are in the spike (S) gene. The six non-synonymous mutations in S correspond to a six-nucleotide deletion (V143–Y145), and five substitutions (H49Y, T95I, F486L, N501T and D614G) (Fig. 4a). With the exception of H49Y, these S mutations originated before the divergence of B.1.641 from the MRCA shared with the Michigan samples. These mutations have previously been observed in animal-derived viral sequences. Not all S mutations were conserved across B.1.641; S:613H and S:L1265I were found only in the human sample, three other non-synonymous mutations were found in either 4658 (T22I and S247G) or 4662 (V705A) white-tailed deer samples, and there was a frameshift in 4662S:L959.

Many non-synonymous mutations had previously been identified in white-tailed deer, including 16 in at least 3/5 of the Ontario deer samples, S:613H and ORF8:Q27* (human sample only), and S:T22I (1/5 Ontario deer samples only, but also noted in Delta-like SARS-CoV-2 from deer in Quebec^26^). However, there were also five conserved non-synonymous mutations that had not been previously observed in white-tailed deer and were relatively rare in GISAID (<1,000 sequences as of 14 March 2022): ORF1a:insertion2038N/MRASD (*n* = 32, including 31 mink from Michigan, United States), ORF1b:V364L (G14557T, *n* = 442, all human sequences), S:F486L (T23020G, *n* = 455), ORF3a:L219V (T26047G, *n* = 886) and ORF10:L37F (C29666T, *n* = 0).

### Mutational signatures of deer adaptation

**Extended Data Fig. 2:**
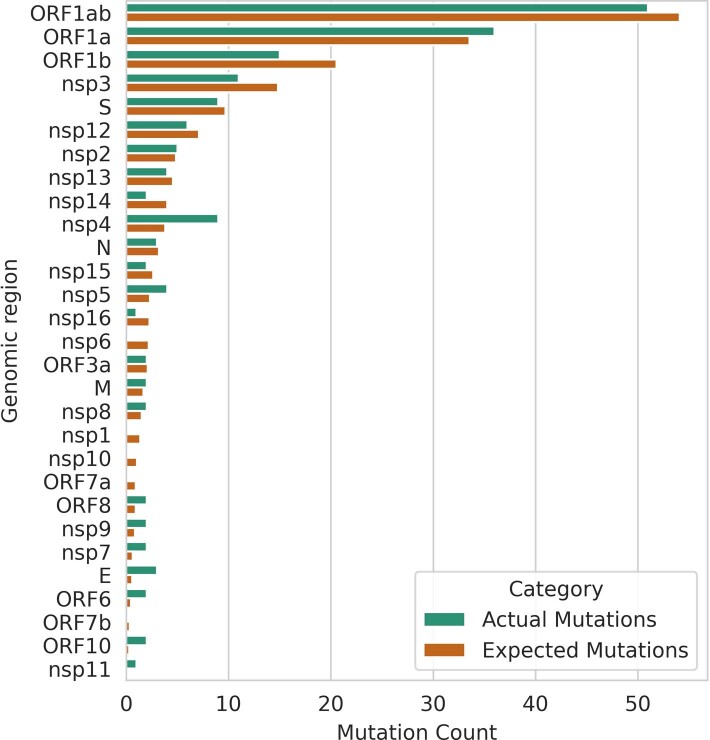
Distribution of conserved mutations in B.1.641. Observed vs expected mutation counts (if uniformly distributed according to gene-length) within shared B.1.641 mutations for each gene/product.

We examined evolutionary parameters to garner insights into selective pressures on B.1.641. We identified a potentially elevated mutation rate (3.7 × 10^−3^ versus 0.9 × 10^−3^ substitutions per site per year; Fig. 4b) relative to other SARS-CoV-2 on the basis of a root-to-tip regression of the global phylogeny (Fig. 2). To characterize signatures of selection within B.1.641 relative to background B.1 samples, we generated codon-alignment phylogenies for S, E, M, N, ORF3a, ORF6 and ORF1ab sequences and applied selection analysis methods from HyPhy^32^. The adaptive branch-site random effects likelihood^36^ and branch-site unrestricted statistical test for episodic diversification^37^ branch-site methods identified no B.1.641 branches with evidence of episodic diversifying positive selection relative to background B.1 branches. Interestingly, the ORF1ab analysis identified significant relaxation of selection amongst the white-tailed deer lineage (*P* = 0.0032). These signatures of neutrality were further supported by the even distribution of conserved mutations in proportion to gene/product length (Extended Data Fig. 2). Together, this suggests sustained viral transmission with minimal immune pressure in a susceptible animal population. However, further investigation into the host response and disease course of SARS-CoV-2 in white-tailed deer is required to confirm these inferences.

**Extended Data Fig. 3:**
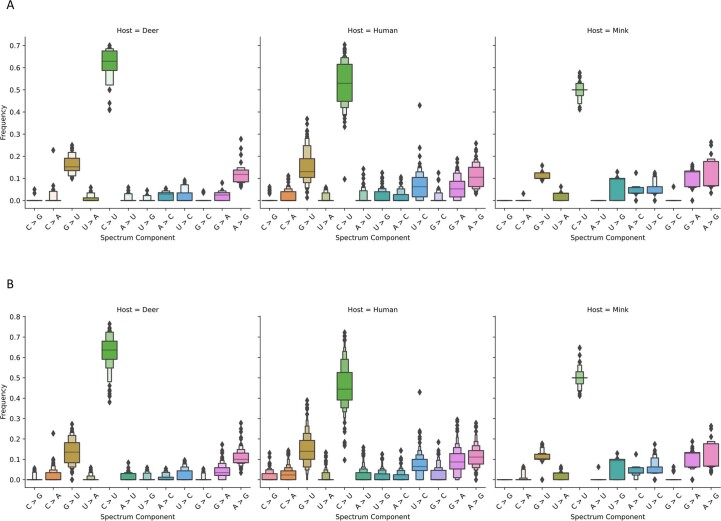
Comparing mutational spectra among hosts. Boxen plots illustrating differences in the mutational spectra among hosts when considering only (**a**) clade 20 C (ndeer = 42, nmink = 91, nhuman = 108) and (**b**) all clades (ndeer = 105, nmink = 432, nhuman = 103). These plots show differences in both the frequency and overall distribution of each mutation within each host species. Samples from each host species were isolated from individuals in North America. The measure of center for each plot is the 50th percentile. The first boxes above and below the 50th percentile represent 50% of the data and each additional box accounts for one-quarter of the remaining data. The total number of boxes and outliers (diamonds) are determined using Tukey’s method.

**Extended Data Fig. 4:**
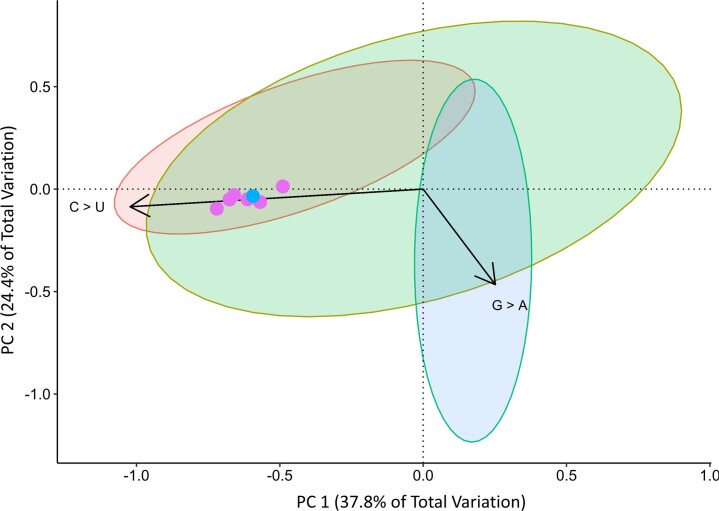
Principal Components Analysis of mutational spectra in Clade 20 C. Principal Components Analysis of the mutational spectra of SARS-CoV-2 genomes isolated from different hosts within Clade 20 C. The first two components account for 62.2% of the variation between the samples. Variation in the spectra along the first principal component associated with changes in the frequency of C > U. Samples appear to be spread along the first component by host-type. Ontario WTD samples (pink) and a human sample from Ontario (blue) appear close together in the projection, suggesting that they share a very similar mutation spectrum.

**Extended Data Fig. 5:**
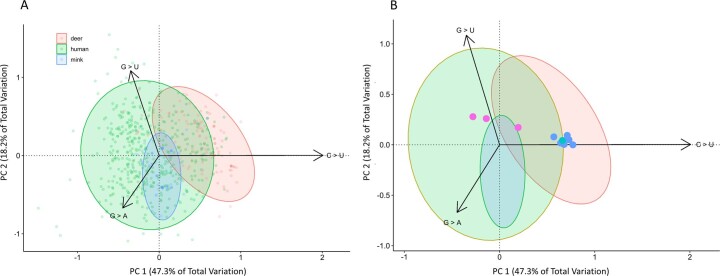
Principal Component Analysis of the mutational spectra from different SARS-CoV-2 variants. The first two principal components account for 65.5% of the variation among the samples. Variation in the spectra along the first principal component are associated with changes in the frequency of C > U mutations. Interestingly, samples along this component are also differentiated by host-type. Variation along the second principal component reflect changes in the frequency of G > U and G > A mutations. (A) The biplot showing a projection of all samples. (B) A simplified version of plot A highlighting the positions of white-tailed deer samples isolated from Ontario (Blue) and Quebec (Pink). A human isolate from Ontario appears in the same area of the plot (teal) as Ontario deer samples. Arrows are scaled to 30% of their original size to create a cleaner plot.

Changes in the mutational signature of SARS-CoV-2 can be used to trace and understand its spread among hosts, and provide insights into mechanistic processes (for example, positive selection, RNA-dependent RNA polymerase activity or host cell modification through RNA editing). An analysis of base substitution frequencies within B.1.641 showed an elevated proportion of mutations involving C > U changes relative to other global, B.1 lineage and animal-derived viral sequences (Fig. 4c and Extended Data Fig. 3). Further investigation using non-parametric distance-based Welch multivariate analysis of variance (dbWMANOVA) found that the mutational spectra between human, deer and mink hosts differs significantly when considering all clades (W*d = 91.04, *P* < 0.001) and within clade 20C, which contains the B.1 lineage and Ontario B.1.641 sequences (W*d = 160.47, *P* < 0.001) (Supplementary Table 3 and Extended Data Fig. 4). Principal component analysis indicated that the majority of this variation (62.2%) corresponded to C > U (PC1) and G > A (PC2) frequencies. Notably, compared with the recently collected deer-derived virus samples from Quebec, the location of B.1.641 suggests that the Ontario lineage has been evolving within deer (Extended Data Fig. 5).

Analyses of genome composition and codon usage bias may provide information on virus evolution and adaptation to host. We assessed similarity of B.1.641 codon usage signatures to other SARS-CoV-2 sequences (samples isolated from Wuhan-Hu-1, Quebec white-tailed deer, and mink from Canada and the United States), cervid viruses (epizootic haemorrhagic disease virus (EHDV), cervid atadenovirus A and elk circovirus) and the *Odocoileus virginianus* genome. No apparent differences were observed in codon usage bias between B.1.641 and other SARS-CoV-2 sequences across the entire coding region of the viral genome. Although some similarity in codon usage bias to cervid atadenovirus A was observed, generally there were clear differences between SARS-CoV-2 and non-SARS-CoV-2 sequences (Supplementary Table 4).

### Virus isolation and S antigenicity

To determine the infectivity of positive samples, virus isolation was carried out using Vero E6 cells expressing human transmembrane protease serine 2 (TMPRSS2) with cathepsin L knocked out. At 4 days post-infection, cytopathic effect of 50% or less of the cell monolayer was observed for four of the samples (4581, 4645, 4658 and 4649) and virus supernatants were harvested. Confirmatory quantitative PCR for SARS-CoV-2 was carried out using 5′ UTR and E RT–PCR targets. The Ct for the four isolated samples (1/7 dilutions), 4581, 4645, 4658 and 4649, were 14.89, 16.39, 12.80 and 13.89 and 16.17, 24.18, 13.06 and 13.91, respectively, for 5′ UTR and E amplicons, respectively. Confirmatory sequencing was carried out successfully for isolates from samples 4581, 4645 and 4658. These showed only minor frequency variations of one single-nucleotide polymorphism (SNP) change (4581: gain of ORF3a Pro42Leu) or two SNP changes (4,658, loss of n.13 T > C and gain of ORF1a p.His3125Tyr) compared with the original swab consensus sequences.

**Fig. 5:**
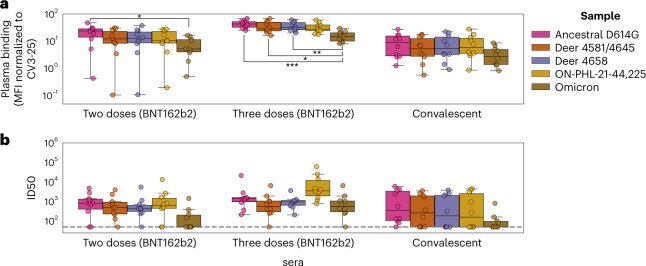
Neutralization of the B.1.641 Ontario white-tailed deer spike. **a**, 293T cells transfected with plasmids encoding the indicated spike variants were incubated with 1:250 diluted plasma from vaccinated (two or three doses of BNT162b2), convalescent or naïve individuals (*n* = 10 for each group) or with the conformationally independent anti-S2 CV3-25 antibody, followed by staining with fluorescently labelled anti-human IgG and flow cytometry analysis. MFI was normalized by surface expression of spike variants on the basis of CV3-25 binding (Supplementary Table 5). Differences in MFI between Omicron and ancestral D614G samples were significant for two-dose (*P* = 0.0284) and three-dose (*P* = 0.001) sera. Similarly, Omicron versus deer 4581/4645 (*P* = 0.0157) and deer 4658 (*P* = 0.0097) were significant for three-dose sera. **b**, Lentiviral pseudotypes encoding luciferase and harbouring the indicated spike variants were incubated with serial dilutions of plasma for 1 h at 37 °C and then used to infect 293T-ACE2. Infection was measured by quantitating luciferase activity 72 h post-infection. Neutralization ID50 for the sera from vaccinated or convalescent individuals was determined using a normalized non-linear regression using GraphPad Prism (Supplementary Table 6). Limit of detection is indicated by a dotted line (ID50 = 50). Distributions across replicates are represented by box plots with a central median value and whiskers showing the 1.5× interquartile range. Significant group differences (from Welch’s one-way ANOVA with Tukey’s post hoc testing) are indicated using brackets and asterisks (*P < 0.05, **P < 0.01, ***P < 0.001).

Considering that S-gene mutations may lead to immune evasion to antibody responses generated by vaccination or previous infection, we measured spike recognition and neutralizing activity of plasma from vaccinated recipients or convalescent individuals to S glycoproteins identified in this study to broach a key element of risk to human health. Cells were transfected with codon-optimized S expression constructs corresponding to the S genes of samples 4581/4645, 4658, ON-PHL-21-44225 or SARS-CoV-2 S:D614G or Omicron (BA.1) and incubated with sera to analyse antibody recognition of S (Fig. 5a). We found that all white-tailed deer S were recognized to a similar extent to the S:D614G by sera from vaccinated or convalescent individuals, while Omicron S was less recognized overall (Fig. 5a and Supplementary Table 5). In addition, lentiviral pseudotypes were incubated with serial dilutions of sera, and neutralization half-maximal inhibitory serum dilution (ID50) was determined (Fig. 5b and Supplementary Table 6). We found that sera from vaccinated recipients, after either two or three doses, and from convalescent individuals efficiently neutralized all B.1.641S proteins, unlike Omicron, which required three doses for neutralization (Fig. 5b). Importantly, we did not observe a difference between the ability of sera to neutralize SARS-CoV-2 D614G or any of the Ontario white-tailed deer SARS-CoV-2. Taken together, these results suggest that the white-tailed deer S-gene mutations do not have substantial antigenic impact on antigenicity.

## Discussion

We identified a divergent lineage of SARS-CoV-2 in white-tailed deer, and report evidence of host adaptation and unsustained deer-to-human transmission. White-tailed deer present many attributes important for a sustainable virus reservoir, including social behaviour, high density, highly transient populations with multiple human–deer interfaces and sylvatic interactions with other animals. A stable reservoir in white-tailed deer means that there is potential for spillover into human and sylvatic wildlife populations over a broad geographic distance. Unlike SARS-CoV-2 in farmed and contained mink, mitigation of onward transmission from wild white-tailed deer to humans is more challenging.

Phylogenetic analysis revealed that B.1.641 shares a relatively recent common ancestor with mink- and human-derived viral sequences from nearby Michigan. This includes specific mutational similarities such as a subset of mink sequences from Michigan exhibiting a rare 12-nucleotide insertion in the ORF1a gene that was also present in B.1.641. Two S-gene mutations, F486L and N501T, have been associated with mustelid (mink or ferret) host adaptations, and N501T has been associated with enhanced ACE2 binding and entry into human (Huh7) cells^7,19,38,39^. Notably, the Ontario white-tailed deer SARS-CoV-2 genomes did not harbour the relatively well-described S:Y453F mutation associated with mink and increased replication and morbidity in ferrets, but reduced replication in primary human airway epithelial cells^40^. Many of the mutations in the B.1.641 genomes have not been described previously or are infrequent and uncharacterized. These deer-derived genomes provide new insights into viral evolution and inferred virus mobility in animal species outside of the human population.

The mutational spectra of SARS-CoV-2 genomes from white-tailed deer, mink and humans vary between hosts, as highlighted by differences within clade 20C. Importantly, this provides evidence supporting the hypothesis that mutational spectra can be used to infer viral host species^9,40^. Furthermore, the frequency of C > U and A > G mutations differed between hosts, which may reflect host cell activity, such as restriction factors (for example, apolipoprotein B messenger RNA editing enzyme, catalytic polypeptide-like or APOBEC family of mRNA editing proteins), RNA editing enzymes (ADAR1) and reactive oxygen species^42–45^. We also observed that the mutational spectrum of B.1.641 is similar to that of other deer. However, this observation does not imply that mutations found in these sequences are related to those found in other deer. Rather, it is likely that the interaction between the viral genome and various host factors will alter the mutation spectra in broad, host-specific ways^9,41^. When placed in the context of the broader literature, our results provide further evidence this lineage of SARS-CoV-2 probably evolved in deer over time.

The absence of detectable positive selection in B.1.641, evidence of relaxation of selection within ORF1ab and the distribution of mutations across the genome contrasts with the signatures of strong selection in the equivalently divergent Omicron VOC. While additional complete deer-derived genomes could enable a more nuanced analysis^46^, it is clear that the evolutionary forces acting within B.1.641 are considerably different to those in Omicron. From these results, and a phylogenetically distant MRCA from 2020, we can infer that the B.1.641 lineage likely diverged in 2020 and has been maintained in wildlife under minimal selection pressure. It is possible that the absence of pre-existing host immunity permitted genetic drift to drive accumulation of neutral mutations (in combination with accumulation of mutations associated with animal adaptation).

**Fig. 6:**
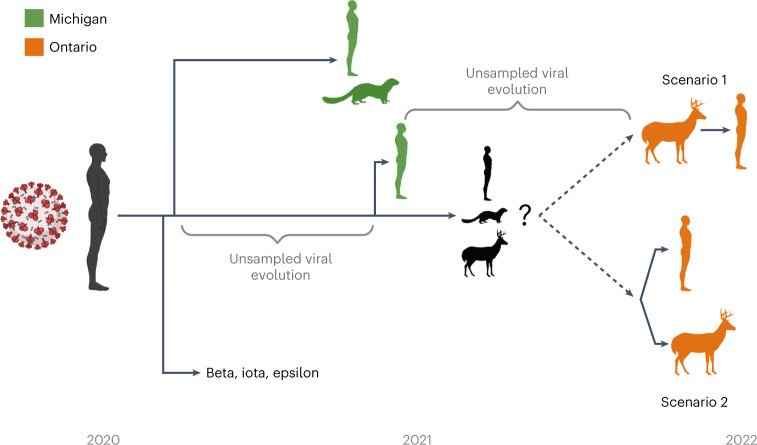
Hypothetical zoonoses and evolution of the B.1.641 lineage. The timeline and approximate relationship between the Beta VOC (bold), Iota/Epsilon former VUIs, and viral samples in white-tailed deer, humans and mink from both Michigan (green) and Ontario (orange) are displayed. As it likely emerged during one of the indicated poorly sampled periods of viral evolution, it is unclear whether the viral ancestor of B.1.641 was from an unknown animal (for example, mink, white-tailed deer or other species) or human reservoir. From this ancestor, there was either a spillback transmission from deer to human (scenario 1) or the emergence of a virus infecting both human and deer (scenario 2).

It is unclear whether the initial spillover occurred directly from humans to deer, or an intermediate host such as mink or other yet undefined species was involved. The long branch length and period of unsampled evolution provide a number of possible scenarios (Fig. 6). The human-derived sample in the B.1.641 lineage lies within a relatively small number of deer-derived samples, rendering the precise relationship between the human- and deer-derived viruses uncertain (78% UFB). B.1.641 could represent a spillover into deer with a human spillback or the emergence of a virus reservoir in another wildlife species infecting both human and deer. However, the epidemiological data, evidence of infectious virus from deer, and the paucity of SARS-CoV-2 surveillance in white-tailed deer relative to human cases suggest spillover in deer followed by deer-to-human transmission is the most likely scenario (Fig. 6, scenario 1).

At this time, there is no evidence of recurrent deer-to-human or sustained human-to-human transmission of B.1.641. However, there has been considerable reduction in human and deer testing and genomic surveillance since the study samples were collected, and we cannot exclude the possibility of sustained transmission within or between these host populations. Enhanced surveillance is critical given human population density and mobility in the region, coupled with white-tailed deer population dynamics. In addition, rapid characterization of this new lineage from biological and epidemiological perspectives is critical to understanding viral transmission, immune evasion and disease in both wildlife and humans. Therefore, we assessed the ability of antibodies elicited following vaccination or infection to recognize and neutralize S and found that the mutations in B.1.641 do not have significant impact on S antigenicity (acknowledging the limited number of plasma tested) (Fig. 5). More work is needed to determine the potential roles of the mutations on spike functions, and to understand the pathogenesis and transmission phenotypes of this virus.

Secondary wildlife reservoirs have the potential to fundamentally alter the ecology of SARS-CoV-2. Our work underscores the need for a broad international One Health focus to identify new intermediate or reservoir hosts capable of driving sustained transmission and divergent viral evolution. An examination of human drivers of spillover and spillback and knock-on effects on wildlife and human health is urgently needed to identify, develop and implement mitigation strategies, beginning with reducing viral activity in humans.

## Methods

### Deer sample collection and study area

Between 1 November and 31 December 2021, adult and yearling free-ranging white-tailed deer were sampled as part of the Ontario Ministry of Natural Resources and Forestry’s (MNRF) annual Chronic Wasting Disease (CWD) surveillance programme. Samples were collected from hunter-harvested deer in Southwestern and Eastern Ontario and included nasal swabs and RPLNs. Samples were collected by staff wearing masks and disposable gloves. Nasal swabs were stored in individual 2 ml tubes with ~1 ml of universal transport medium (Sunnybrook Research Institute (SRI)), and RPLN tissues were stored dry in 2 ml tubes. After collection, samples were immediately chilled on ice packs then transferred to a −20 °C freezer where they were held for up to 1 week. Samples were then transferred to a −80 °C freezer where they were held until analysis. Location, date of harvest, and demographic data (age/sex) were recorded for each animal when available.

### PCR screening and detection

RNA extractions and PCR testing of samples collected from deer were performed at the SRI in Toronto, Ontario. RNA extractions were conducted with 140 µl of nasal swab sample spiked with Armored RNA enterovirus (Asuragen; https://www.asuragen.com) via the Nuclisens EasyMag using Generic Protocol 2.0.1 (bioMérieux Canada) according to the manufacturer’s instructions; RNA was eluted in 50 µl. Tissue samples were thawed, weighed, minced with a scalpel and homogenized in 600 µl of lysis buffer using the Next Advance Bullet Blender (Next Advance) and a 5 mm stainless steel bead at 5 m s^−1^ for 3 min. RNA from 30 mg tissue samples was extracted using Specific Protocol B 2.0.1 via Nuclisens EasyMag; RNA was eluted in 50 µl. RT–PCR was performed using the Luna Universal Probe One-Step RT–qPCR kit (New England BioLabs, NEB; https://www.neb.ca). A SARS-CoV-2 5′ UTR and E specific multiplex RT–PCR were used for RNA detection^47^. Quantstudio 3 software (Thermo Fisher Scientific; https://www.thermofisher.com) was used to determine the cycle threshold (Ct). All samples were run in duplicate, and samples with Ct <40 for both RT–PCR targets and Armored RNA enterovirus in at least one replicate were considered presumed positive. For tissue samples, the presence of inhibitors was assessed by a 1:5 dilution of one of the replicates. Samples were considered inconclusive if no Armored enterovirus was detected or if only one RT–PCR target was detected and re-extracted for additional analysis. Samples were considered indeterminate if inconclusive after re-extraction or if no original material was left. Presumed positive samples were further analysed for human RNAse P to rule out potential human contamination^9^. Original material from presumed positive samples detected at SRI were sent to the Canadian Food Inspection Agency (CFIA) for confirmatory PCR testing. The MagMax CORE Nucleic Acid Purification Kit (Thermo Fisher Scientific) and the automated KingFisher Duo Prime magnetic extraction system was used to extract total RNA spiked with Armored RNA enterovirus. The enteroviral armored RNA was used as an exogenous extraction control. A SARS-CoV-2 E and nucleocapsid (N) specific multiplex RT–PCR was used for confirmatory RNA detection^7^. Master mix for qRT–PCR was prepared using TaqMan Fast Virus 1-step Master Mix (Thermo Fisher Scientific) according to the manufacturer’s instructions. Reaction conditions were 50 °C for 5 min, 95 °C for 20 s and 40 cycles of 95 °C for 3 s then 60 °C for 30 s. Runs were performed by using a 7500 Fast Real-Time PCR System (Thermofisher, ABI). Samples with Ct <36 for both RT–PCR targets were considered positive.

### WGS

WGS was performed at both SRI and CFIA using independent extractions and sequencing methods. At SRI, DNA was synthesized from extracted RNA using 4 μl LunaScript RT SuperMix 5× (NEB) and 8 μl nuclease free water, and was added to 8 μl extracted RNA. Complementary DNA synthesis was performed under the following conditions: 25 °C for 2 min, 55 °C for 20 min, 95 °C for 1 min and holding at 4 °C.

The ARTIC V4 primer pool (https://github.com/artic-network/artic-ncov2019) was used to generate amplicons from the cDNA. Specifically, two multiplex PCR tiling reactions were prepared by combining 2.5 μl cDNA with 12.5 μl Q5 High-Fidelity 2× Master Mix (NEB), 6 μl nuclease-free water and 4 μl of respective 10 μM ARTIC V4 primer pool (Integrated DNA Technologies). PCR cycling was then performed in the following manner: 98 °C for 30 s followed by 35 cycles of 98 °C for 15 s and 63 °C for 5 min.

PCR reactions were then both combined and cleaned using 1× ratio sample purification beads (Illumina) at a 1:1 bead to sample ratio. The amplicons were quantified using the Qubit 4.0 fluorometer using the 1× dsDNA High Sensitivity (HS) Assay Kit (Thermo Fisher Scientific) and sequencing libraries prepared using the Nextera DNA Flex Prep kit (Illumina) as per the manufacturer’s instructions. Paired-end (2 × 150 bp) sequencing was performed on a MiniSeq with a 300-cycle reagent kit (Illumina) with a negative-control library with no input SARS-CoV-2 RNA extract.

WGS performed at CFIA used extracted nucleic acid quantified using the Qubit RNA HS Assay Kit on a Qubit Flex Fluorometer (Thermo Fisher Scientific). Eleven microlitres or 200 ng of total RNA was subject to DNase treatment using the ezDNase enzyme (Thermo Fisher Scientific) according to the manufacturer’s instructions. DNase-treated RNA was then used for library preparation and target sequence capture according to the ONETest Coronaviruses Plus Assay protocol (Fusion Genomics^48^). The enriched libraries were quantified using the Qubit 1× dsDNA HS Assay Kit on a Qubit Flex Fluorometer (Thermo Fisher Scientific) and subsequently pooled in equimolar amounts before fragment analysis on 4200 TapeStation System using the D5000 ScreenTape Assay (Agilent). The final pooled library was sequenced on an Illumina MiSeq using a V3 flowcell and 600 cycle kit (Illumina).

Human specimens are received at Public Health Ontario Laboratory for routine SARS-CoV-2 diagnostic testing (RT–PCR) from multiple healthcare settings, including hospitals, clinics and coronavirus disease 2019 (COVID-19) assessment centres. The human sample (ON-PHL-21-44225) was sequenced at Public Health Ontario Laboratory using an Illumina-based ARTIC V4 protocol (10.17504/protocols.io.b5ftq3nn), similar to the deer sequencing methods. Briefly, cDNA was synthesized using LunaScript reverse transcriptase (NEB). Amplicons were generated with premixed ARTIC V4 primer pools (Integrated DNA Technologies). Amplicons from the two pools were combined, purified with AMPure XP beads (Beckman Coulter) and quantified. Genomic libraries were prepared using the Nextera XT DNA Library Preparation Kit (Illumina), and genomes were sequenced as paired-end (2 × 150 bp) reads on an Illumina MiSeq instrument.

### Genomic analysis

Paired-end illumina reads from ARTIC V4 and Fusion Genomics sequencing were initially analysed separately with the nf-core/viralrecon Nextflow workflow (v2.3) (refs. ^49–51^) that ran: FASTQC (v0.11.9) (ref. ^52^) read-level quality control, fastp (v0.20.1) (ref. ^53^) quality filtering and adapter trimming, Bowtie2 (v2.4.2) (ref. ^54^) read mapping to Wuhan-Hu-1 (MN908947.3) (ref. ^55^) SARS-CoV-2 reference, Mosdepth (v0.3.1) (ref. ^56^)/Samtools (v.1.12) (ref. ^57^) read mapping statistics calculation, iVar (v1.3.1) (ref. ^58^) ARTIC V4 primer trimming, variant calling and consensus generation; SnpEff (v5.0) (ref. ^59^)/SnpSift (v4.3t) (ref. ^60^) for variant effect prediction and annotation; and Pangolin (v3.1.20) (ref. ^61^) with PangoLEARN (2022-01-05), Scorpio (v0.3.16) (ref. ^62^), and Constellations (v.0.1.1) was used for PANGO lineage^63^ assignment. iVar primer trimmed soft-clipped read alignments were converted to hard-clipped alignments with fgbio ClipBam (http://fulcrumgenomics.github.io/fgbio/). Reads from hard-clipped BAM files were output to FASTQ files with ‘samtools fastq’. nf-core/viralrecon was re-run in amplicon mode without iVar primer trimming on the combined Fusion Genomics and ARTIC V4 primer trimmed FASTQ reads to generate the variant calling, variant effect and consensus sequence results used in downstream analyses. Additional quality-control steps to check for negative-control validity, drop-out, sample cross-contamination and excess ambiguity were performed using ncov-tools v1.8.0 (ref. ^64^). The mutations identified by the Nextclade (v1.10.2) (ref. ^65^) with 2022-01-05 database and xlavir (v0.6.1) report were manually searched in outbreak.info’s ‘Lineage | Mutation Tracker’ (on 2 February 2022) (ref. ^66^) to get information on the prevalence of observed mutations globally and within Canada. Mutations were also investigated for presence in specific lineages including VOCs, Michigan mink samples and other animal samples. Finally, mutations were searched in GISAID (on 2 February 2022) to tally the number of non-human hosts each mutation had been observed in.

Some limitations in genome quality and coverage existed that may have resulted in failure to detect additional mutations. All B.1.641 samples had missing terminal domains and contained internal regions with no or low coverage when sequenced using the ARTIC v4 amplicon scheme. This is a widespread issue that may explain the rarity of the 3′ proximal ORF10:L37F in GISAID. Significantly in our samples this meant there was no or <10× coverage in all five deer-derived sequences from ~27000 to 27177 (drop-out of ARTICv4 amplicons 90-91), which includes regions of the M gene. However, by combining the ARTIC v4 sequencing with additional sequencing using probe-based enrichment we were able to compensate for this drop-out and generate high coverage and completeness (<100 positions with no coverage in all deer and <100 positions with <10× coverage in 3/5 deer genomes; Supplementary Table 7).

### Phylogenetics

To evaluate possible sampling biases due to the poorly defined and diverse B.1 and B.1.311 lineages and select closely related publicly available sequences for further phylogenetic analysis, a phylogenetic placement analysis based on UShER (https://genome.ucsc.edu/cgi-bin/hgPhyloPlace)^34^ was performed using the 7,217,299 sample tree (derived from UShER placement of GISAID, GenBank, COG-UK and CNCB onto 13-11-20 sarscov2phylo ML tree) via the SHUShER web-portal (shusher.gi.ucsc.edu). Phylogenetic analyses were performed using CFIA-NCFAD/scovtree Nextflow workflow (v1.6.0) (https://github.com/CFIA-NCFAD/scovtree/) with the consensus sequences contextualized with closely related sequences identified by UShER and randomly sampled representative sequences from major WHO SARS-CoV-2 clades from GISAID^12^ (downloaded 10 February 2022). This workflow generated a multiple sequence alignment using Nextalign CLI (v1.10.1) (ref. ^65^) and inferred an ML phylogeny using IQ-TREE (v2.2.0_beta)^67^ using the general time-reversible (GTR) model for visualisation with Phylocanvas^68^ via shiptv (v0.4.1) (https://github.com/CFIA-NCFAD/shiptv) and ggtree^69^. Divergence times for the inferred global ML topology were estimated using BEAST v1.10.4 (ref. ^70^). This analysis used a coalescent model with constant population size, a Hasegawa–Kishono–Yano substitution model with four discrete gamma categories, and a log-normal distributed strict molecular clock rate of 9.5 × 10^−4^ substitutions per site per year (based on a tip-to-root regression performed using TempEst^71^). Internal node heights and root node height were sampled by BEAST over 50 million MCMC generations (recorded every 1,000 iterations) before collation using BEAST’s LogCombiner. The final maximum clade credibility tree was generated using BEAST’s TreeAnnotator with node heights set to median values before final visualization in FigTree and Inkscape.

A subset of 157 taxa from an ancestral clade of B.1.641 were selected from the global phylogenetic tree shown in Fig. 2 to generate the phylogenetic tree shown in Fig. 3. Multiple sequence alignment of this subset of sequences was performed with MAFFT (v7.490) (ref. ^72^). An ML phylogenetic tree was inferred with IQ-TREE (v2.2.0_beta) using the GTR model and 1,000 UFB replicates^73^. Nextclade (v1.10.2) analysis was used to determine amino acid mutations and missing or low/no coverage regions from the sample genome sequences. Amino acid mutation profiles were determined relative to the B.1.641 samples, discarding mutations that were not present in any of the Ontario samples. Taxa with duplicated amino acid mutation profiles were pruned from the tree, keeping only the first observed taxa with a duplicated profile.

Recombination analyses were performed using 3Seq (v1.7) (ref. ^30^) and Bolotie (e039c01) (ref. ^31^). Specifically, 3Seq was executed with B.1.641 sequences and the most recent example of each lineage found in Canada and closest samples in GISAID in subtree (*n* = 595). Bolotie was executed using the B.1.641 sequences and two datasets, the provided pre-computed 87,695 probability matrix and a subsample of the earliest and latest example of each lineage in GISAID with all animal-derived samples and closest UShER samples (*n* = 4,688). Additionally, HyPhy’s^32^ (v2.5.31) genetic algorithm recombination detection method^33^ was applied to local alignment and ML phylogeny (Fig. 3) for all possible sites. Phylogenies were inferred using IQTree for segments either side of the identified putative breakpoint, and the B.1.641 clade and local topology was unchanged. Sequence statistics such as C > T rate were directly calculated from nextclade results (v1.10.2 with 2022-01-05 database). Additional figures were generated and annotated using BioRender^74^ and Inkscape^75^.

A phylogenetic approach with HyPhy^32^ (v2.5.31) was used to investigate signatures of selection within B.1.641 relative to the wider B.1 background lineage. To ensure necessary genomic completeness for codon alignment, all B.1 sequences in GISAID (as of 8 March 2022) were filtered to those with <0.1% N with full date information. Genomes with 0% N were removed to avoid biases from consensus workflows that replace undetermined sequence with reference genome. From this, all animal-derived (49 mink and 1 cat) sequences and 100 randomly sampled human B.1 sequences were extracted (*n* = 150). Finally, the WH0-1 reference genome was added to this alignment along with the five complete Ontario deer-derived genomes, associated human sequence, and the two most closely related Michigan human samples (MI-MDHSS-SC23517 and M-MDHSS-SC22669). Virulign^76^ (v1.0.1) was then used to generate codon alignments for E, M, N, S, ORF1ab, ORF3a, ORF6, ORF7a, ORF7b, ORF8 and ORF10 genes relative to the Wuhan-Hu-1 (MN908947.3) reference. ML phylogenies were inferred for these alignments using raxml-ng (v1.0.2) (ref. ^77^) with the GTR model and three parsimony-based starting trees. These phylogenies were manually inspected and rooted on Hu-1, and the B.1.641 branches were labelled using phylowidget^37,78^. Genes for which the phylogeny did not have a resolved B.1.641 clade (ORF7a and ORF7b) or a viable codon alignment without any internal stop codons (ORF8 and ORF10) were excluded from further analyses. For each gene, signatures of positive selection were evaluated using HyPhy’s adaptive branch-site random effects likelihood (aBSREL) method^36^ and signatures of gene-wide episodic diversification were evaluated using the branch-site unrestricted statistical test for episodic diversification (BUSTED) method^37^ with ten starting points. Finally, evidence of intensification or relaxation of selection was investigated using the RELAX method^79^ with ten starting points and synonymous rate variation. Additional code for divergence dating, recombination and selection analyses can be found under 10.5281/zenodo.7086599.

### Analysis of mutational spectrum

The mutational spectra were created using a subset of 3,645 sequences used to create the high-quality global phylogeny. Included in this dataset are the seven unique deer samples from Ontario (samples 4538, 4534, 4662, 4649, 4581, 4645 and 4658; the five high-quality genomes plus two genomes with lower coverage), three white-tailed deer samples from Quebec (samples 4055, 4022 and 4249) and one human sample from Ontario (ON-PHL-21-44225), and any remaining human, mink and deer sequences from North America. The counts for each type of nucleotide change, with respect to the reference strain, were compiled and used to create a 12-dimensional vector. This subset was then filtered to remove sequences with fewer than 15 nucleotide changes. The counts were converted into the mutation spectrum by simply dividing each count by the sum of the counts in each sample^10,41^. As the mutation spectrum summarizes how host factors act upon the genome of SARS-CoV-2, it can potentially be used as an independent source of evidence supporting the evolution of the virus in a different host^10,41^. To investigate this, we conducted experiments using a dbWMANOVA^80^. If a significant difference between hosts was detected, a pairwise distance-based Welch *t*-test was used to identify which pair of hosts differed^81^. This approach was used because it is more robust on unbalanced and heteroscedastic data^80,81^. In the first experiment, samples from all lineages were used. The second experiment used only the subset of samples belonging to Nextstrain clade 20C (which contains the B.1.641 sequences). An analysis using the entire Pangolin B.1 lineage was not appropriate since this lineage also includes Nextstrain clades 20A and 20B and the inclusion of these samples could potentially obfuscate important patterns since the results would have to be interpreted in a context wider than necessary.

### Codon usage analysis

Consensus sequences of SARS-CoV-2 samples from this and previous studies and additional sequences gathered from public databases were used. The sequences include the reference SARS-CoV-2 Wuhan-Hu-1 (NCBI NC_045512), SARS-CoV-2 mink/Canada/D/2020 (GISAID EPI_ISL_717717), SARS-CoV-2 mink/USA/MI-20-028629-004/2020 (GISAID EPI_ISL_2834697), *C*ervid atadenovirus A 20-5608 (NCBI OM470968)^82^, EHDV serotype 2/strain Alberta (NCBI AM744997 - AM745006), epizootic haemorrhagic disease virus, EHDV serotype 1/New Jersey (NCBI NC_013396 - NC_013405), EHDV 6 isolate OV208 (NCBI MG886400 - MG886409) and elk circovirus Banff/2019 (NCBI MN585201) (ref. ^83^) were imported into Geneious (v.9.1.8) (ref. ^84^). Annotations for the coding sequences of SARS-CoV-2 samples were transferred from the reference sequence SARS-CoV-2 Wuhan-Hu-1 (NC_ 045512) using the Annotate from Database tool. The coding sequences were extracted using the Extract Annotations tool for all viral sequences. An annotated file of the coding sequences for the *Odocoileus virginianus texanus* isolate animal Pink-7 (GCF_002102435.1) genome was downloaded from NCBI (https://ftp.ncbi.nlm.nih.gov/genomes/all/annotation_releases/9880/100/GCF_002102435.1_Ovir.te_1.0/). Coding sequences were input into CodonW (http://codonw.sourceforge.net/) with settings set to concatenate genes and output to file set to codon usage. Codon usage indices were set to the effective number of codons (ENc), GC content of gene (G + C), GC of silent third codon position (GC3s), silent base composition, number of synonymous codons (L_sym), total number of amino acids (L_aa), hydrophobicity of protein (Hydro) and aromaticity of protein (Aromo).

### Virus isolation

For virus isolation, T25 flasks were seeded to confluency 1 day before infection with cathepsin L knock-out Vero E6 cells overexpressing TMPRSS2. The following day, swab samples were vortexed and spun down and 200 μl of the swab samples medium was combined with 16 μg ml^−1^ working concentration of TPCK-treated trypsin (NEB), 2× A/A/p/s antifungal/antibiotic solution (Wisent) and a 0.1% working concentration of BSA (Thermo Fisher Scientific) and added to the cell monolayer after removal of the medium. Samples were subjected to a 45 min adsorption with rocking every 5 min, after which the inoculum was removed and discarded and the monolayer was either washed once with 2 ml of D1 to remove blood cells present in the samples (4581, 4649 and 4676) or not washed (4645, 4658 and 4662) and 5 ml of DMEM with 1% FBS and antibiotics was added to the flask and incubated at 37 °C with 5% CO_2_. At 4 days post-infection, samples with visible cytopathic effect (partial, 50% or less rounded or detached cells) were harvested followed by collection and centrifugation at 4,000*g* for 10 min at 20 °C. The harvested supernatants were aliquoted and stored at −80 °C or inactivated and removed from the CL3 laboratory and RNA was extracted with the QIAamp Viral RNA Mini Kit (Qiagen), and stored at −20 °C until downstream analyses were carried out. All infectious work was performed under biosafety level 3 conditions.

### Codon-optimized spike constructs, cells, sera and antibodies

Expression constructs of S mutants corresponding to samples 4581/4645 (S:H49Y, S:T95I, S:Δ143-145InsD, S:F486L, S:N501T, S:D614G), 4658 (S:T22I, S:H49Y, S:T95I, S:Δ143-145InsD, S:S247G, S:F486L, S:N501T, S:D614G) and ON-PHL-21-44225 (S:H49Y, S:T95I, S:Δ143-145InsD, S:F486L, S:N501T, S:Q613H, S:D614G) were generated by overlapping PCR as described previously^85^. S:D614G and S Omicron (BA.1) constructs were described elsewhere^86^. All constructs were cloned in pCAGGS and verified by Sanger sequencing.

HEK293T cells (ATCC) were cultured in DMEM supplemented with 10% FBS (Sigma), 100 U ml^−1^ penicillin, 100 µg ml^−1^ streptomycin and 0.3 mg ml^−1^ l-glutamine (Invitrogen) and maintained at 37 °C, 5% CO_2_ and 100% relative humidity.

Serum samples were obtained from consenting participants in several cohort studies with sample collection and sharing for this analysis approved by the Sinai Health System Research Ethics Board (#22-0030-E). Plasma of SARS-CoV-2 naïve, naïve-vaccinated (28–40 days after two or three doses of BNT162b2) and unvaccinated SARS-CoV-2 Delta previously infected donors was collected (Supplementary Table 8), heat inactivated for 1 h at 56 °C, aliquoted and stored at −80 °C until use. The conformation-independent monoclonal anti-S2 CV3-25 from a convalescent individual was described and produced as described previously^87,88^. The goat anti-human IgG conjugated with Alexa Fluor-647 was purchased from Invitrogen (A21445).

### Spike binding assays

**Extended Data Fig. 6:**
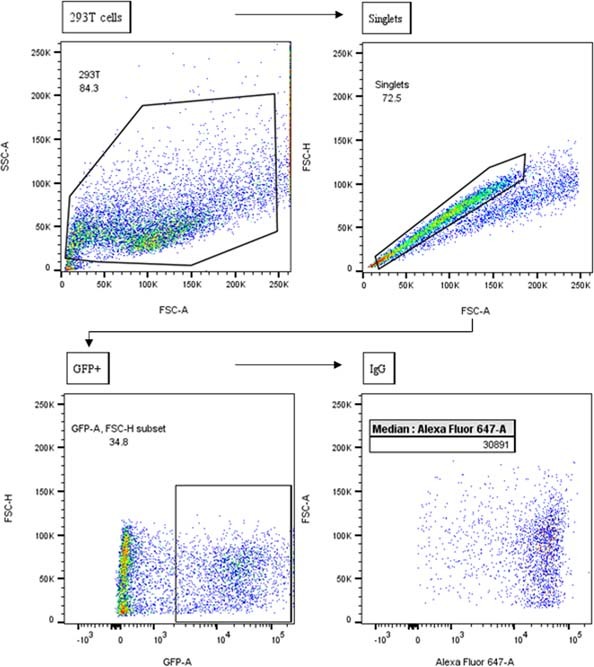
Flow cytometry gating strategy for IgG plasma binding analysis. HEK 293 T cells were identified in forward scatter (FSC) and side scatter (SSC). Single cells (Singlets) were selected for analysis using FSC-A and FSC-H. Cells transfected with SARS-CoV-2 spike protein plasmid were gated (GFP+), negative and auto-fluorescent cells were excluded. Median fluorescence intensity for IgG (AlexaFluor 647-A) was analyzed in GFP + cells. Data was analyzed using FlowJo software.

Hek293T cells seeded in a 10 cm Petri dish at 70% confluency were transfected with 10 µg of SARS-CoV-2 spike protein plasmid, 1 µg of lentiviral vector bearing green fluorescent protein (GFP) (PLV-eGFP) (gift from Pantelis Tsoulfas, Addgene plasmid number 36083) (ref. ^89^) using Jetprime transfection reagent (Polyplus, catalogue number 101000046) according to the manufacturer’s instructions. At 16 h post-transfection, the cells were stained with sera samples (1:250 dilution) for 45 min at 37 °C. Alexa Fluor-647-conjugated goat anti-human IgG (H + L) was used to detect plasma binding of the treated cells following 1 h incubation at room temperature. Samples were washed once with PBS, fixed in 1% paraformaldehyde and acquired using BD LSR Fortessa Flow cytometer (BD Biosciences). The seropositivity threshold was defined on the basis of mean fluorescence intensity (MFI) for naïve samples plus three standard deviations. The data were normalized by surface expression on the basis of the MFI of the monoclonal antibody CV3-25 (5 μg ml^−1^). The data analysis was performed using FlowJo 10.8.1 (Extended Data Fig. 6). For each set of sera, binding was compared across samples using Welch’s (unequal variance) one-way ANOVA procedure and a post-hoc Tukey’s honestly significant difference test (using a family-wise error rate of 0.05) via the statsmodel library (v0.14.0) (ref. ^90^).

### Pseudotype production and neutralization assays

HEK293T seeded in 10 cm dishes were co-transfected with lentiviral packaging plasmid psPAX2 (gift from Didier Trono, Addgene number 12260), lentiviral vector pLentipuro3/TO/V5-GW/EGFP-Firefly Luciferase (gift from Ethan Abela, Addgene number 119816) and plasmid encoding the indicated S construct at a 5:5:1 ratio using jetPRIME transfection reagent according to the manufacturer’s protocol. Twenty-four hours post-transfection, media were changed, and supernatants containing lentiviral pseudotypes were harvested 48 h post-transfection, filtered with a 0.45 µM filter and stored at −80 °C until use.

HEK293T stably expressing human ACE2 (293T-ACE2, kind gift of Hyeryun Choe, Scripps Research^91^) were seeded in poly-d-lysine-coated 96-well plates. The next day, supernatants containing lentiviral pseudotypes were incubated with sera (serially diluted by five-fold, from 1:50 to 156,250) for 1 h at 37 °C and then added to cells in the presence of 5 µg ml^−1^ polybrene. Seventy-two hours later, media were removed, and cells were rinsed in phosphate-buffered saline and lysed by the addition of 40 µl passive lysis buffer (Promega) followed by one freeze–thaw cycle. A Synergy Neo2 Multi-Mode plate reader (BioTek) was used to measure the luciferase activity of each well after the addition of 50–100 µl of reconstituted luciferase assay buffer (Promega) as per the manufacturer’s protocol. Neutralization ID50 was calculated using Graphpad Prism and represents the plasma dilution that inhibits 50% of pseudotype transduction in 293T-ACE2. For each set of sera, neutralization was compared across samples using Welch’s (unequal variance) one-way ANOVA procedure and a post-hoc Tukey’s honestly significant difference test (using a family-wise error rate of 0.05) via the statsmodel library (v0.14.0) (ref. ^90^).

### Reporting summary

Further information on research design is available in the Nature Research Reporting Summary linked to this article.

## Supplementary information

Supplementary Information Legends for Supplementary Tables 1–9.

Reporting Summary

Supplementary Data Supplementary Fig. 1. UShER-based phylogenetic tree with Ontario white-tailed deer-derived high quality (*n* = 5) and partial (*n* = 2) genomes and the 2022-02-21 USCS UShER build (shared in Newick format).

Supplementary Data A Microsoft Excel Workbook containing Supplementary Tables 1–9.

## Data Availability

All genomic sequence data are publicly available data through GISAID (https://gisaid.org/), and SRA accession numbers are provided in the supplementary material (Supplementary Table 1). Computer code and analysis scripts can be accessed at 10.5281/zenodo.7086599. All other data are available in the supplementary material (Supplementary Tables 1–9).

## Code availability

All computer code and analysis scripts used in the manuscript are archived at https://github.com/fmaguire/on_deer_spillback_analyses/ and can be accessed at 10.5281/zenodo.7086599.
