# Supplementary material for: Divergent SARS-CoV-2 variant emerges in white-tailed deer with deer-to-human transmission
Source: Nat Microbiol. 2022 Nov 10;7(12):2011–24. doi: 10.1038/s41564-022-01268-9 (PMC9712111; doi:10.1038/s41564-022-01268-9)
Supplement: Supplementary file 1 — Legends for Supplementary Tables 1–9. [file 41564_2022_1268_MOESM1_ESM.pdf]

nature microbiology

Article https://doi.org/10.1038/s41564-022-01268-9

Divergent SARS-CoV-2 variant emerges in white-tailed deer with deer-to-human transmission

In the format provided by the authors and unedited

Supplementary Table Legends

Supplementary tables are shared in a Microsoft Excel workbook called "Supplementary_Tables.xlsx". Table captions are provided below in the order in which they appear referenced in the manuscript and in the workbook.

Table S1: Metadata associated with the 300 white-tailed deer samples and 1 human sample screened for SARS-CoV-2. Sample IDs used in the manuscript are the last 4 digits of the WILD-CoV Sample ID. * Samples not selected for sequencing with combined capture enrichment and ARTICv4 approach based on initial quality of ARTICv4 sequencing. + Human-derived sample sequenced by PHOL as part of public health surveillance. RPLN: Retrophyarngeal Lymph Nodes, RT-PCRs at SRI (UTR and E gene Ct<40) and CFIA (E and N2 gene Ct<36). Samples were considered positive (PVE), negative (NVE), or indeterminate (IND). For samples sent for confirmatory testing, positive (+) and negative (-) results are indicated in brackets from SRI and CFIA, respectively. Notably, results from SRI for two samples could only be considered inconclusive (+?) as remaining original material was depleted for confirmatory testing at CFIA.

Table S2: Summary of mutations within B.1.641, the Ontario white-tailed deer lineage (with associated human sequence) and their distribution across GISAID sequences, VOC, animal-derived viral sequences, and related Michigan mink sequences.

Table S3: Results of a distance-based Welch MANOVA investigating differences in the mutation spectrum between hosts within Nextstrain Clade 20C. 999 permutations were used to generate one-sided p-values.

Table S4: Summary of codon usage bias analysis results across SARS-CoV-2 from white-tailed deer (including B.1.641, the Ontario lineage) and other cervid viruses.

Table S5: Plasma binding assay data (MFI normalised to CV3-25).

Table S6: Neutralisation assay data (ID50).

Table S7: Poor coverage regions in the Ontario B.1.641 lineage sequenced with combined ARTIC V4 and capture enrichment (ONETest) data.

Table S8: Description of the cohort and sera that was used for plasma binding and neutralisation assays.

Table S9: Acknowledgement table for sequences used from GISAID for phylogenetic and mutational signature analyses.
