# Supplementary material for: Divergent SARS-CoV-2 variant emerges in white-tailed deer with deer-to-human transmission
Source: Nat Microbiol. 2022 Nov 10;7(12):2011–24. doi: 10.1038/s41564-022-01268-9 (PMC9712111; doi:10.1038/s41564-022-01268-9)
Supplement: Supplementary file 2 — Reporting Summary [file 41564_2022_1268_MOESM2_ESM.pdf]

nature portfolio

Corresponding author(s): Bradley Pickering, Samira Mubareka, and Jeff Bowman

Last updated by author(s): Sep 20, 2022

Reporting Summary

Nature Portfolio wishes to improve the reproducibility of the work that we publish. This form provides structure for consistency and transparency in reporting. For further information on Nature Portfolio policies, see our Editorial Policies and the Editorial Policy Checklist.

Statistics

For all statistical analyses, confirm that the following items are present in the figure legend, table legend, main text, or Methods section.

n/a	Confirmed	
☐	☒	The exact sample size (*n*) for each experimental group/condition, given as a discrete number and unit of measurement
☒	☐	A statement on whether measurements were taken from distinct samples or whether the same sample was measured repeatedly
☐	☒	The statistical test(s) used AND whether they are one- or two-sided<br>*Only common tests should be described solely by name; describe more complex techniques in the Methods section.*
☐	☒	A description of all covariates tested
☐	☒	A description of any assumptions or corrections, such as tests of normality and adjustment for multiple comparisons
☐	☒	A full description of the statistical parameters including central tendency (e.g. means) or other basic estimates (e.g. regression coefficient) AND variation (e.g. standard deviation) or associated estimates of uncertainty (e.g. confidence intervals)
☐	☒	For null hypothesis testing, the test statistic (e.g. *F*, *t*, *r*) with confidence intervals, effect sizes, degrees of freedom and *P* value noted<br>*Give P values as exact values whenever suitable.*
☒	☐	For Bayesian analysis, information on the choice of priors and Markov chain Monte Carlo settings
☒	☐	For hierarchical and complex designs, identification of the appropriate level for tests and full reporting of outcomes
☒	☐	Estimates of effect sizes (e.g. Cohen's *d*, Pearson's *r*), indicating how they were calculated

Our web collection on statistics for biologists contains articles on many of the points above.

Software and code

Policy information about availability of computer code

Data collection	All computer code and analysis scripts used in the manuscript are archived here (https://github.com/fmaguire/on_deer_spillback_analyses/) and can be accessed at DOI: 10.5281/zenodo.7086599.
Data analysis	All computer code and analysis scripts used in the manuscript are archived here (https://github.com/fmaguire/on_deer_spillback_analyses/) and can be referenced as DOI: 10.5281/zenodo.7086599. In addition, we used Quantstudio 3 for RNA detection, GraphPad Prism 9 for ID50, and FlowJo 10.8.1 for flow cytometry analysis.

For manuscripts utilizing custom algorithms or software that are central to the research but not yet described in published literature, software must be made available to editors and reviewers. We strongly encourage code deposition in a community repository (e.g. GitHub). See the Nature Portfolio guidelines for submitting code & software for further information.

Data

Policy information about availability of data

All manuscripts must include a data availability statement. This statement should provide the following information, where applicable:

- Accession codes, unique identifiers, or web links for publicly available datasets
- A description of any restrictions on data availability
- For clinical datasets or third party data, please ensure that the statement adheres to our policy

All genomic sequence data are publicly available data through GISAID (https://gisaid.org/) and SRA accession numbers are provided in the supplementary material (Table S1). Computer code and analysis scripts can be accessed at DOI: 10.5281/zenodo.7086599. All other data are available in the supplementary materials (Tables S2 to S7).

Field-specific reporting

Please select the one below that is the best fit for your research. If you are not sure, read the appropriate sections before making your selection.

☒ Life sciences ☐ Behavioural & social sciences ☐ Ecological, evolutionary & environmental sciences

For a reference copy of the document with all sections, see nature.com/documents/nr-reporting-summary-flat.pdf

Life sciences study design

All studies must disclose on these points even when the disclosure is negative.

Sample size	Sample sizes for SARS-CoV-2 genomic sequences were determined by prevalence of active SARS-CoV-2 infection of white-tailed deer in the study area, and our subsequent ability to generate high quality whole genome sequences. We sampled all hunter-harvested deer that were available to us, and we sequenced all of the available deer-derived virus in the sample of deer. Sample sizes for experiments using sera were determined by availability of sera.
Data exclusions	Some genomes with low coverage were excluded from some analyses. This is fully described in the manuscript.
Replication	We replicated whole genome sequencing by completing sequencing at two separate labs using independent extractions and sequencing methods. All deer-derived genomes were sequenced multiple times using this approach. Other experiments (e.g., virus isolation and neutralization) were not replicated.
Randomization	We systematically sampled hunter-harvested white-tailed deer from two regions of Ontario, Canada that were being surveyed for other purposes (i.e., a Chronic Wasting Disease (CWD) surveillance program). We took advantage of the ongoing CWD program to conduct SARS-CoV-2 surveillance and sequenced all of the available deer-derived virus in these study areas. For experiments with sera, human participants were randomly selected within treatment groups.
Blinding	Blinding was not relevant to much of our study as we were sampling hunter-harvested deer for SARS-CoV-2. For virus neutralization assays however, investigators were blinded to sera sources for data collection and analysis.

Reporting for specific materials, systems and methods

We require information from authors about some types of materials, experimental systems and methods used in many studies. Here, indicate whether each material, system or method listed is relevant to your study. If you are not sure if a list item applies to your research, read the appropriate section before selecting a response.

Materials & experimental systems

n/a	Involved in the study	
☐	☒	Antibodies
☐	☒	Eukaryotic cell lines
☒	☐	Palaeontology and archaeology
☐	☒	Animals and other organisms
☐	☒	Human research participants
☒	☐	Clinical data
☒	☐	Dual use research of concern

Methods

n/a	Involved in the study	
☒	☐	ChIP-seq
☐	☒	Flow cytometry
☒	☐	MRI-based neuroimaging

Antibodies

Antibodies used	Secondary antibody: Goat anti-Human IgG (H+L) Secondary Antibody, Alexa Fluor™ 647 (at a 1:250 dilution). Vendor: ThermoFisher Scientific. Catalog number: A-21445. Lot number: 2339821. CV3-25 was described by Jennewein et al. Cell Rep., 36, 110210. Details are provided in the methods.
Validation	From the ThermoFisher catalog: To minimize cross-reactivity, these goat anti-human IgG (H+L) whole secondary antibodies have been affinity purified and cross-adsorbed against mouse, rabbit, and bovine serum prior to conjugation. Anti-Human secondary antibodies are affinity-purified antibodies with well-characterized specificity for human immunoglobulins and are useful in the detection, sorting or purification of its specified target. For experiments in this study, mock-transfected cells that were not expressing Spike were used as negative controls.

Eukaryotic cell lines

Policy information about cell lines

Cell line source(s)	HEK293T cells (from ATCC) and HEK293T-Ace2 from 293T-ACE2 (provided by Hyeryun Choe, Scripps Research). While these

Cell line source(s)	latter cells were originally from ATCC, they have been modified to overexpress human ACE2, and have been described by Moore et al. J Virol., 78, 10628–10635). Details are fully described and cited in the methods.
Authentication	The cell lines were not authenticated.
Mycoplasma contamination	All cell lines tested negative for mycoplasma contamination.
Commonly misidentified lines (See ICLAC register)	No commonly misidentified cell lines were used in the study.

Animals and other organisms

Policy information about studies involving animals; ARRIVE guidelines recommended for reporting animal research

Laboratory animals	*For laboratory animals, report species, strain, sex and age OR state that the study did not involve laboratory animals.*
Wild animals	We sampled hunter-harvested white-tailed deer from two regions of Ontario, Canada that were being surveyed for other purposes (i.e., a Chronic Wasting Disease (CWD) surveillance program). We took advantage of the ongoing CWD program to conduct SARS-CoV-2 surveillance and sampled tissues or took nasal swabs for deer that had already been harvested by hunters.
Field-collected samples	*For laboratory work with field-collected samples, describe all relevant parameters such as housing, maintenance, temperature, photoperiod and end-of-experiment protocol OR state that the study did not involve samples collected from the field.*
Ethics oversight	No ethical approval was required as all deer used in the study were harvested by licensed hunters in Ontario, Canada.

Note that full information on the approval of the study protocol must also be provided in the manuscript.

Human research participants

Policy information about studies involving human research participants

Population characteristics	Inpatients and outpatients recovered from or vaccinated for COVID-19. Covariate information (e.g., age and sex) is provided in the supplementary material (Table S8).
Recruitment	Retrospective
Ethics oversight	Sinai Health System Research Ethics Board (#22-0030-E), Sunnybrook HSC Research Ethics Board (#SUN-2218), University of Ottawa Research Ethics Board (#H-01-22-7842).

Note that full information on the approval of the study protocol must also be provided in the manuscript.

Flow Cytometry

Plots

Confirm that:

☒ The axis labels state the marker and fluorochrome used (e.g. CD4-FITC).

☒ The axis scales are clearly visible. Include numbers along axes only for bottom left plot of group (a 'group' is an analysis of identical markers).

☒ All plots are contour plots with outliers or pseudocolor plots.

☒ A numerical value for number of cells or percentage (with statistics) is provided.

Methodology

Sample preparation	Transfected HEK293T cells were stained with sera samples (1:250 dilution) for 45min at 37°C. Alexa Fluor-647-conjugated goat anti-human IgG (H+L) was used to detect plasma binding of the treated cells following 1 hour incubation at room temperature. Samples were washed once with PBS, fixed in 1% paraformaldehyde before flow cytometry analysis. Details are provided in the methods.
Instrument	BD LSR Fortessa Flow cytometer (BD Biosciences)
Software	Data collection: BD FACSDiva, data analysis: FlowJo
Cell population abundance	N.A. no sorting of populations involved in this study.
Gating strategy	Preliminary gating for live cells by SSC/FSC, and further secondary gating for single cells. Gating of Spike-expressing and GFP-cotransfected cells was then performed using mock-transfected cells as negative controls. The gating strategy is shown in Extended data Figure 6.

☒ Tick this box to confirm that a figure exemplifying the gating strategy is provided in the Supplementary Information.
